# Accuracy of artificial intelligence-based detection of dental crowding and spacing on cone-beam computed tomography

**DOI:** 10.1186/s12903-026-09405-0

**Published:** 2026-07-25

**Authors:** Natalia Kazimierczak, Łukasz Wanczura, Tomasz Kulczyk, Tomasz Jankowski, Agnieszka Jankowska, Emilia Zielińska, Emilia Czarnecka, Małgorzata Dittrich, Wiktoria Biegała, Zbigniew Serafin, Wojciech Kazimierczak

**Affiliations:** 1 Kazimierczak Clinic, Dworcowa 13/u6a, Bydgoszcz, 85-009 Poland; 2 Faculty of Medicine, Collegium Medicum, Nicolaus Copernicus University in Torun, Jagiellońska 13-15, Bydgoszcz, 85-067 Poland; 3 Department of Diagnostic Imaging, Poznan University of Medical Sciences, Poznań, 61- 701 Poland; 4 Private Practice Dental Clinic Jankowscy, Czerwonego Krzyża 24, Żary, 68-200 Poland; 5 Faculty of Medicine, Bydgoszcz University of Science and Technology, Kaliskiego 7, Bydgoszcz, 85-796 Poland; 6 Department of Radiology and Diagnostic Imaging, Collegium Medicum, Nicolaus Copernicus University in Torun, Jagiellońska 13-15, Bydgoszcz, 85-067 Poland

**Keywords:** Artificial intelligence, Cone-beam computed tomography, Dental crowding, Dental spacing, Diagnostic accuracy, Little’s irregularity index, Orthodontic diagnosis

## Abstract

**Background:**

To evaluate the accuracy of an artificial intelligence (AI) system for detecting anterior dental crowding and spacing on cone-beam computed tomography (CBCT) and to characterise clinically relevant patterns of diagnostic failure.

**Methods:**

In this retrospective diagnostic accuracy study, CBCT scans from 88 patients were analysed. AI-generated diagnoses at the tooth-pair level were compared with a reference standard based on a modified Little’s Irregularity Index (mLII) that incorporates signed contact-point displacement. Diagnostic performance — sensitivity, specificity, positive and negative predictive values, and Cohen’s κ — was assessed at the tooth-pair level (*n* = 875) across five thresholds (± 0.5 to ± 2.5 mm). Arch-level performance was assessed using the conventional Little’s Irregularity Index (LII). Statistical significance was set at α = 0.05, and all estimates are reported with 95% confidence intervals.

**Results:**

At the tooth-pair level, sensitivity for crowding ranged from 28.8% to 42.0%, whereas specificity remained high (81.9–88.9%). Agreement with the reference standard was fair (maximum κ = 0.247). A consistent blind spot was identified at the maxillary central-incisor pair (11–21), where no crowded cases were detected despite confirmed irregularities. Spacing detection was poor (sensitivity 10–25%). At the arch level, detection improved with increasing severity of irregularity (38.8–75.0%) but remained incomplete and was accompanied by a high false-positive rate (41.2% in perfectly aligned arches).

**Conclusions:**

The AI system showed limited sensitivity and only fair agreement for detecting anterior crowding and spacing on CBCT. Clinically relevant irregularities, including those at the anterior midline, may be missed. Because crowding and spacing are most reliably assessed from clinical examination and intraoral records rather than from CBCT, AI-based CBCT assessments should be interpreted with caution and cannot replace clinician evaluation.

## Background

Malocclusion is among the most common abnormalities of the human dentition and is generally regarded as a developmental disturbance rather than a disease [1, 2]. Dental crowding — a discrepancy between tooth size and arch dimensions — is one of its most prevalent forms and occurs irrespective of sex, race, or socioeconomic status [1, 3, 4]. It arises when tooth size and arch dimensions are poorly coordinated, and its aetiology is multifactorial, reflecting the combined influence of genetic and environmental factors on arch morphology [4]. Beyond its aesthetic impact, crowding has functional consequences: it complicates daily oral hygiene and may thereby contribute to caries and periodontal disease [1, 5].

Accurate assessment of the arch perimeter, and of the space available for tooth alignment, has long been a central concern in orthodontics. Traditional approaches range from visual estimation to the brass-wire and caliper methods [6, 7]. Although the brass-wire and caliper techniques are valued for their speed and simplicity, both have important limitations: the brass-wire method depends heavily on operator judgement, whereas the caliper method approximates the arch as a series of straight-line segments and therefore tends to underestimate the true arch perimeter [6]. More valid and reproducible measurement methods are consequently needed to support stable treatment outcomes and a sound root–alveolar relationship [8].

Cone-beam computed tomography (CBCT) overcomes several of these limitations by providing high-resolution, three-dimensional (3D) imaging of the maxillofacial region without the anatomical superimposition inherent in two-dimensional (2D) radiography [9–11]. CBCT-derived models allow reliable measurement of tooth dimensions and arch relationships and support complex analyses, such as the relationship between crowding and apical-base size [8, 12]. For patients with impacted teeth, facial asymmetry, or airway concerns, CBCT also provides diagnostic information that is essential for comprehensive orthodontic planning [10, 11]. It should be emphasised, however, that CBCT entails ionising radiation and is not the primary modality for routine assessment of crowding or spacing, which are most often evaluated clinically or from intraoral scans and dental casts.

Orthodontics is undergoing a rapid digital transformation, and AI is increasingly being integrated to improve diagnostic efficiency and reduce observer variability [13–16]. Deep-learning models, and convolutional neural networks (CNNs) in particular, have achieved high performance in 2D cephalometric analysis [17, 18], and emerging multimodal systems offer new possibilities for assessing crowding severity directly from photographs [16]. Applying AI to 3D CBCT data is more demanding, owing to the high computational requirements and the complexity of the training datasets involved [14, 15]. Nevertheless, AI-driven systems hold promise for standardised, evidence-based evaluation that could support clinical decisions such as predicting treatment outcomes or determining the need for extractions [13–16].

Commercial AI platforms are already being adopted in routine practice, and their outputs may directly influence diagnosis and treatment planning [19, 20]. Independent, task-specific validation is therefore essential before such tools are relied upon. To date, no study has evaluated AI-based detection of crowding and spacing on CBCT at the level of the individual tooth pair.

We hypothesised that AI-based detection of anterior crowding and spacing on CBCT would show substantial agreement with the expert-derived reference standard. The aim of this study was to evaluate the accuracy of an AI system for detecting anterior crowding and spacing on CBCT at the tooth-pair level and to identify clinically relevant limitations of current AI-based orthodontic assessment.

## Methods

### Study design

This was a retrospective diagnostic accuracy study that compared an AI platform with an expert-consensus reference standard for the orthodontic diagnosis of dental crowding and spacing. Reporting follows the Standards for Reporting of Diagnostic Accuracy Studies (STARD) 2015 guidelines [21]. All CBCT examinations had been acquired for explicit clinical indications as part of routine patient care; none was performed for research purposes.

### Patient sample selection and data curation

Patient records were retrieved retrospectively from the clinical databases and the Picture Archiving and Communication System (PACS) of three private dental clinics in Poland. We identified consecutive patients aged 13–55 years who had undergone CBCT imaging with a field of view (FOV) encompassing both the maxillary and mandibular dentition between January 2024 and April 2025. CBCT had been ordered by the treating dentists on the basis of explicit clinical indications, including assessment of alveolar bone dimensions and arch morphology, evaluation of impacted teeth, assessment of skeletal asymmetry, and pre-surgical planning.

### Inclusion and exclusion criteria

Eligible scans had a FOV sufficient to encompass the maxilla, mandible, and full dentition, and were obtained from patients in permanent or late mixed dentition. Scans were excluded if they were of diagnostically unacceptable quality (e.g. motion artefacts, beam hardening, or other distortions) or if the patient had a history of significant craniofacial trauma, a congenital craniofacial syndrome, or previous orthognathic surgery. From an initial cohort of 90 patients (Clinic A, 60; Clinic B, 15; Clinic C, 15), two cases from Clinic A were excluded for motion artefacts, yielding a final sample of 88 patients.

### Sample size

The sample size was determined a priori for the primary outcome, namely the sensitivity of AI crowding detection at the tooth-pair level. Using the Buderer formula for diagnostic accuracy studies [22], the required number of diseased (crowded) tooth pairs was estimated as:

$$\mathrm{n} = \mathrm{Z} ^{2} \times \mathrm{SE} \times \left( 1 - \mathrm{Se} \right) / \mathrm{W} ^{2}$$

where Z = 1.96 (corresponding to 95% confidence), Se is the expected sensitivity (0.35, based on preliminary data), and W is the desired half-width of the confidence interval for sensitivity (0.10). This yielded a minimum of 88 crowded tooth pairs. Assuming a crowding prevalence of approximately 40% at the ± 1.0 mm threshold, at least 220 tooth pairs (≥ 22 patients) were required. To support robust subgroup analyses, the sample was enlarged to 88 patients, which provided 875 evaluable tooth pairs, including 225 crowded pairs at the ± 1.0 mm threshold.

### Imaging protocols and de-identification

#### CBCT acquisition

CBCT images were acquired at the three clinics using three units. Clinic A used a Hyperion X9 PRO (MyRay, Imola, Italy; 90 kV, 36 mA, FOV 13 × 10 cm); Clinic B used a Cranex 3Dx (Soredex, Tuusula, Finland; 89 kV, 4.5 mA, 8 s, FOV 13 × 15 cm); and Clinic C used a Hyperion X9 PRO (MyRay; 90 kV, 36 mA, FOV 13 × 10 cm). All volumes were reconstructed with an isotropic voxel size of 0.3 mm.

#### Data anonymisation and blinding

All DICOM files were anonymised, and each patient was assigned a unique alphanumeric code (e.g. A001, C006) that was used for all subsequent analyses. No clinical data were analysed.

### Index test: automated analysis by the Diagnocat AI platform

Anonymised DICOM series for all 88 scans were uploaded to the cloud-based Diagnocat AI platform (version 1.0; Diagnocat Ltd., San Francisco, CA, USA) between 11 December 2025 and 19 January 2026. For each case, the platform automatically generated a diagnostic report, which was exported for analysis. The CBCT AI Report included the platform’s diagnosis of crowding or spacing for adjacent tooth pairs. The platform did not provide exportable contact-point coordinates or editable segmentation masks; therefore, the present validation was limited to the categorical crowding/spacing outputs available in the clinical report. These findings were standardised and entered into a spreadsheet covering all permanent positions (18–48) for each patient; each pair was assigned one of three possible AI labels: no diagnosis, crowding, or spacing.

### Reference standard

#### Measurement software

All CBCT datasets were imported into dedicated DICOM visualisation software (syngo.via, version 8.9 [x64]; Siemens Healthineers GmbH, Erlangen, Germany), which supports multiplanar reconstruction (MPR) and calibrated linear measurement of CBCT volumes.

#### Rationale for a modified Little’s Irregularity Index

Little’s Irregularity Index (LII) is the most widely validated measure of anterior dental crowding; it has excellent reproducibility (intraclass correlation coefficient [ICC] > 0.97) and is accepted by Cochrane as a valid outcome measure [23, 24]. The index sums the linear displacements of the anatomical contact points between adjacent teeth from canine to canine in the occlusal plane, producing a single arch-segment score in which 0 mm denotes perfect alignment. El-Zanaty et al. showed that the LII derived from CBCT models is as accurate as OrthoCAD measurements [25]. Two features of the original index nonetheless limit its direct use here: it yields only absolute values, and so cannot distinguish crowding from spacing; and it produces a single aggregate score per arch, whereas the AI platform reports a diagnosis for each tooth pair.

We therefore applied a modified Little’s Irregularity Index (mLII) that retains the validated measurement principle of the LII — contact-point displacement on standardised axial CBCT slices — while adding two extensions:

- A signed convention, where positive values denote crowding (overlap or displacement between contact points) and negative values denote spacing (visible gap between contact points without contact between adjacent teeth);
- Analysis at the individual tooth-pair level, enabling direct comparison with the per-pair output of the AI platform. In cases of perfect alignment (contact points coinciding with no gap), a value of 0 mm was assigned.

The mLII is thus a methodological extension rather than a new index: the underlying geometric measurement is identical to that of the validated LII, and the modifications simply preserve directional information and match the per-pair output format of the AI.

#### Scope of application

The mLII was applied to the anterior segment (canine to canine) of each arch, yielding five pair measurements per arch: 13–12, 12–11, 11–21, 21–22, and 22–23 in the maxilla, and 43–42, 42–41, 41–31, 31–32, and 32–33 in the mandible. The premolar and molar regions were excluded because the original LII has not been validated for posterior segments.

#### CBCT image standardisation

A standardised 3D orientation protocol adapted from Wang et al. was applied to every dataset [8]. The axial reference plane was oriented to pass through the incisal edges of the central incisors and the mesiobuccal cusps of the right first molars, with verification on the left side. The measurement slice was taken parallel to this plane, at the level of the maximum mesiodistal diameter of the central incisors near the incisal edge; separate axial slices were used for the maxillary and mandibular arches when necessary. When beam-hardening artefacts obscured a contact point, adjacent slices (± 0.3–0.6 mm) were examined.

### Measurement protocol

#### Step 1 — contact-point identification

The mesial and distal contact points of each of the six anterior teeth were identified. In well-aligned teeth, the contact point was the point of closest proximity or actual contact between adjacent surfaces. In displaced, rotated, or overlapping teeth, it was defined as the most prominent mesial or distal point of the crown at the measurement slice, whether or not physical contact with the adjacent tooth occurred.

#### Step 2 — signed displacement

For each adjacent pair, displacement was measured as the shortest linear distance, parallel to the occlusal plane, between the distal contact point of the more mesial tooth and the mesial contact point of the more distal tooth. The sign was assigned as follows:

- Positive (+) for crowding (contact points displaced by overlap, rotation, or labiolingual displacement; magnitude equal to the displacement distance);
- Zero (0 mm) for perfect alignment (coincident contact points, with neither overlap nor gap);
- Negative (−) for spacing (a visible gap, with absolute magnitude equal to the diastema width).

This signed convention enables the mLII to capture both crowding and spacing on a single continuous scale, allowing direct comparison with the trichotomous AI output.

### Diagnostic classification

#### Tooth-pair level (primary analysis, mLII)

Each of the five mLII measurements per arch was classified independently according to predefined thresholds: crowding, mLII ≥ + 1.0 mm; spacing, mLII ≤ − 1.0 mm; and no discrepancy, − 1.0 mm < mLII < + 1.0 mm. Because a single displacement measurement cannot determine which tooth contributes more to the discrepancy, the crowding or spacing label was attributed to both teeth in the pair. A sensitivity analysis was performed at thresholds of ± 0.5, ± 1.0, ± 1.5, ± 2.0, and ± 2.5 mm.

#### Arch-segment level (secondary analysis, LII)

The sum of the five absolute mLII values per arch gave a total anterior irregularity score, categorised according to Little’s grading: 0 mm, perfect; 1–3 mm, minimal; 4–6 mm, moderate; 7–9 mm, severe; and ≥ 10 mm, very severe. This score was compared with whether the AI flagged any anterior tooth within the arch. The dual-level scheme allows both a granular per-pair analysis that matches the AI output format and a conventional arch-segment analysis grounded in the validated LII.

### Examiners and reliability assessment

Measurements were performed independently by two calibrated examiners — a radiologist and an orthodontist, each with more than 10 years of experience in oral imaging — who were blinded to the AI results and to each other’s measurements. Calibration on ten training cases was led by a senior radiologist (> 10 years of CT/CBCT experience) before the main study. Inter-examiner reliability was assessed using the ICC (two-way random effects, absolute agreement, single measures; ICC [2,1]). Intra-examiner reliability was assessed by having one examiner repeat the measurements on 20 randomly selected cases after an interval of at least two weeks.

### Consensus reference standard

For each tooth pair, the reference contact-point displacement was defined as the arithmetic mean of the two examiners’ measurements, and the diagnostic classification was obtained by applying the predefined thresholds to this mean. The arch-level reference score was the sum of the five absolute mean displacements per arch. Averaging the examiners’ measurements minimises random measurement error and is consistent with established practice for diagnostic accuracy studies that lack a single gold standard [26].

### Statistical analysis

Statistical significance was set at α = 0.05, and all estimates are reported with 95% confidence intervals (CIs). *Inter-reader reliability was assessed using the intraclass correlation coefficient (ICC)*,* specified as a two-way random-effects*,* absolute-agreement*,* single-measures model (ICC [2,1])*,* and interpreted as poor (< 0.50)*,* moderate (0.50–0.75)*,* good (0.75–0.90)*,* or excellent (> 0.90)* [27]*. Intra-reader reliability was assessed by ICC for one readerr who repeated the measurements on 20 randomly selected cases after at least two weeks.*

Diagnostic accuracy was evaluated at two levels. At the tooth-pair level (primary analysis), sensitivity, specificity, positive predictive value (PPV), negative predictive value (NPV), and overall accuracy were derived from 2 × 2 contingency tables, computed separately for crowding and for spacing. Wilson score 95% CIs were calculated for sensitivity, specificity, PPV, and NPV. Cohen’s κ was used to quantify overall three-class agreement (crowding, spacing, no discrepancy) and interpreted according to conventional benchmarks (≤ 0.20, slight; 0.21–0.40, fair; 0.41–0.60, moderate; 0.61–0.80, substantial) [28]. These diagnostic accuracy measures are descriptive estimates reported with their confidence intervals; no separate null-hypothesis significance test was applied to them. The sensitivity analysis was repeated across all five mLII thresholds. At the arch-segment level (secondary analysis), the total LII score was compared with whether the AI flagged at least one anterior tooth as crowded, and detection rates were stratified by Little’s severity grade. In addition to sensitivity, specificity, and predictive values, we computed the F1 score and the positive and negative likelihood ratios (LR + = sensitivity/[1 − specificity]; LR− = [1 − sensitivity]/specificity) for crowding and spacing detection at each mLII threshold. 95% confidence intervals for the likelihood ratios were calculated using the log method of Simel et al. [29].

All analyses were performed in Python 3.11 using the SciPy, scikit-learn, and pandas libraries.

## Results

### Study sample and reference-standard reliability

The final sample comprised 88 patients (55 women, 33 men) with a mean age of 27.2 ± 9.5 years (median 27.0; range 13–51). Mean age was 27.8 ± 10.4 years (range 13–51) among women and 26.1 ± 7.9 years (range 13–49) among men. The three clinics contributed 58, 15, and 15 patients, respectively.

The Diagnocat platform generated 498 diagnoses (445 crowding, 53 spacing) in 65 of the 88 patients; the remaining 23 patients received no AI-flagged anterior discrepancy. For the reference standard, 875 mLII measurements were obtained (five were unavailable because of missing teeth); two patients had no measurable anterior discrepancy and contributed only to the arch-level analysis.

**Fig. 1:**
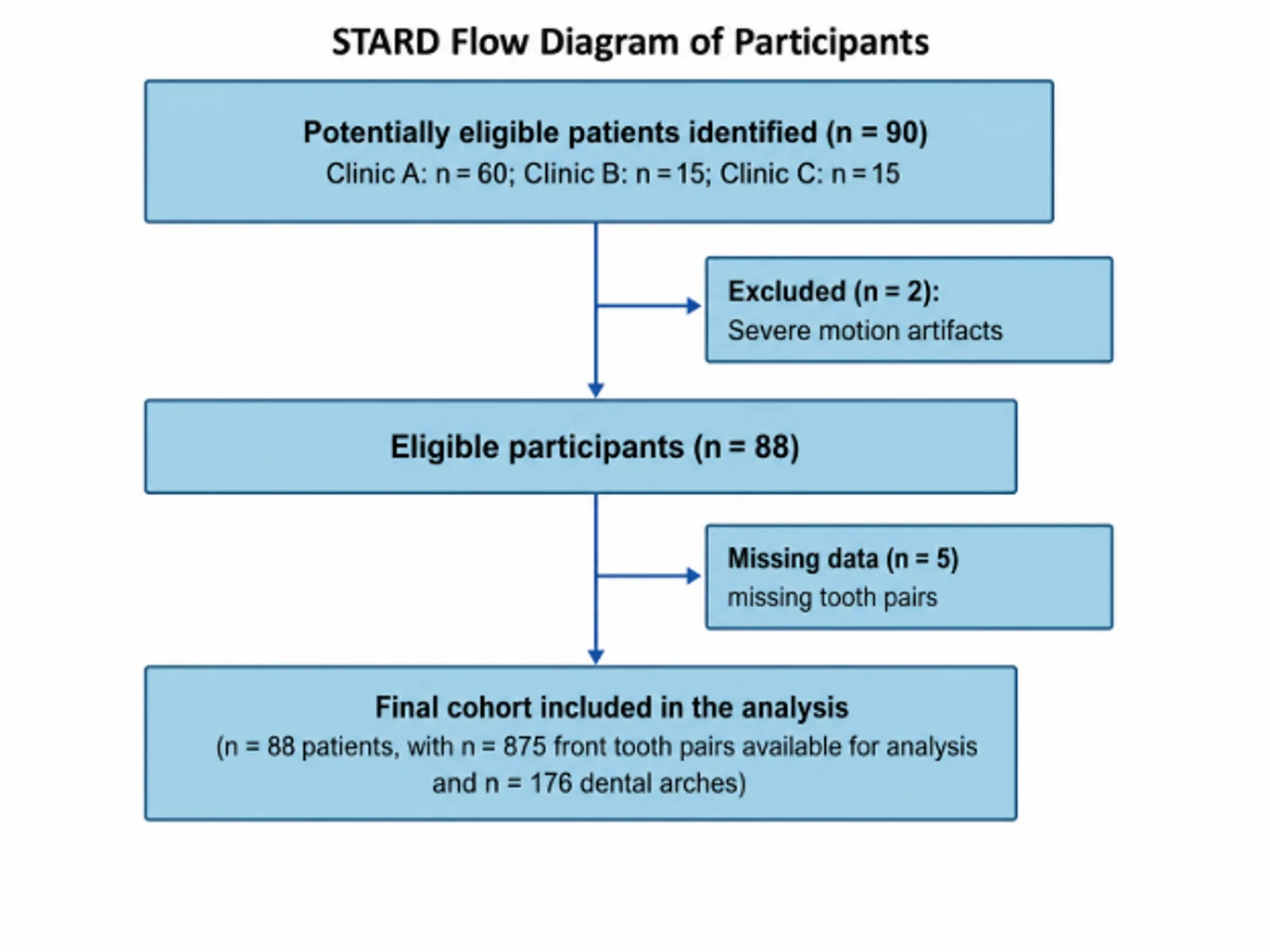
STARD flow diagram of the study material

Inter-reader reliability was excellent (overall ICC [2,1] = 0.946). Individual tooth-pair ICCs ranged from 0.756 (42–41) to 0.975 (21–22), with 9 of 10 pairs exceeding 0.89. The mean absolute inter-reader difference was 0.345 mm (SD 0.332 mm), and only 2.9% of paired measurements differed by more than 1.0 mm. Intra-reader reliability was likewise excellent, with ICC values of 0.965 and 0.967 for Readers I and II, respectively. The STARD flow diagram is shown in Fig. 1.

### Tooth-pair-level crowding detection

**Fig. 2:**
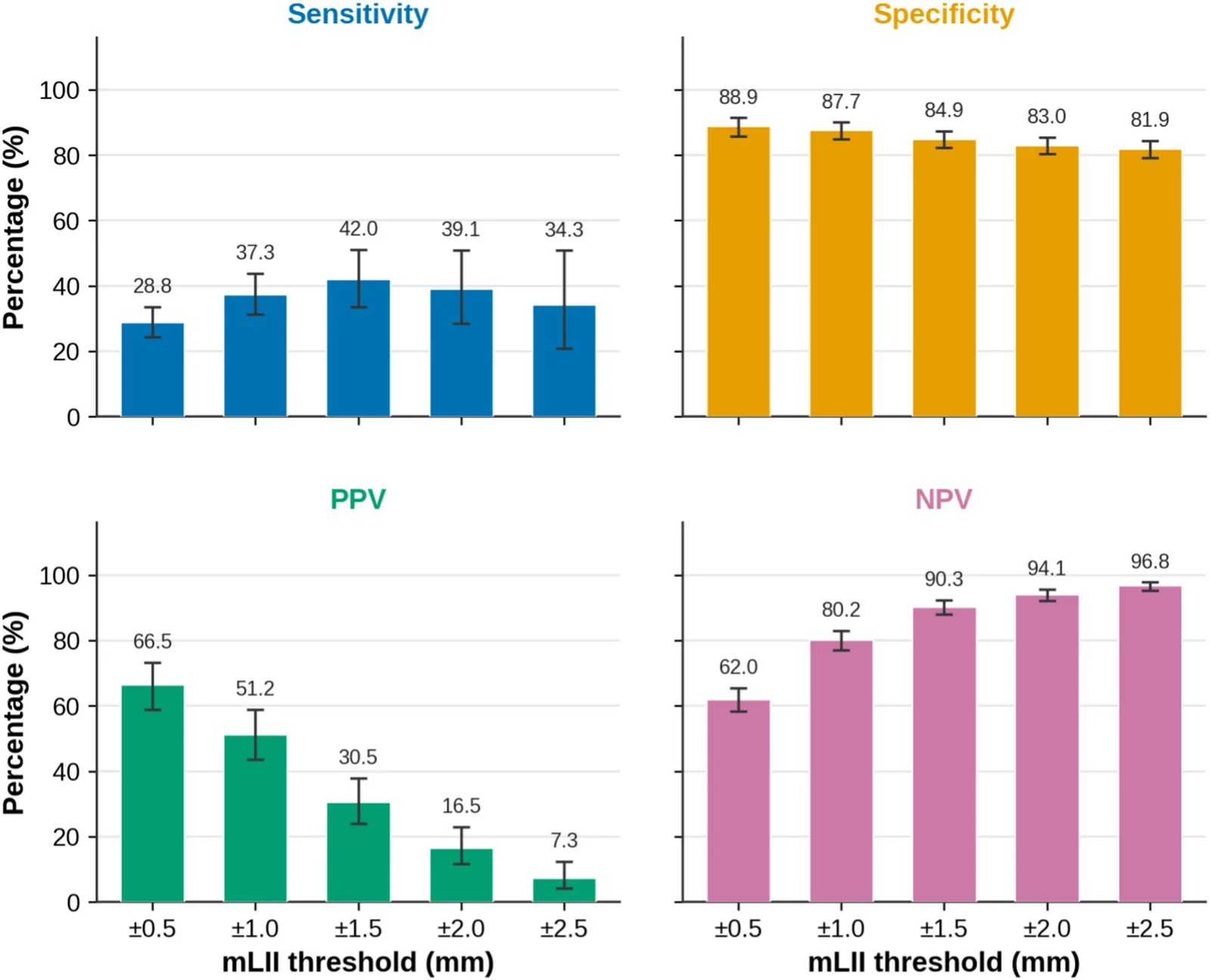
Diagnostic accuracy of the Diagnocat AI platform for anterior crowding detection at the tooth-pair level across five modified Little’s Irregularity Index (mLII) thresholds (± 0.5, ± 1.0, ± 1.5, ± 2.0, and ± 2.5 mm). Bars represent point estimates for sensitivity, specificity, positive predictive value (PPV), and negative predictive value (NPV); error bars indicate 95% Wilson score confidence intervals

Diagnostic accuracy across the five mLII thresholds is summarised in Table 1. Sensitivity ranged from 28.8% (± 0.5 mm) to 42.0% (± 1.5 mm), meaning that the AI missed 58–71% of crowded tooth pairs. Specificity was high at every threshold (81.9–88.9%). PPV fell sharply as the threshold became more stringent, from 66.5% at ± 0.5 mm to 7.3% at ± 2.5 mm. The highest value of Cohen’s κ was 0.247, at ± 1.0 mm, indicating only fair agreement (Fig. 2). The F1 score followed the same pattern as Cohen’s κ, peaking at the ± 1.0 mm threshold (F1 = 0.432) and declining at more lenient and more stringent cut-offs (0.401 at ± 0.5 mm to 0.121 at ± 2.5 mm). The positive likelihood ratio was modest across all thresholds (LR + 1.89–3.03), indicating that a positive AI flag only slightly increased the probability of crowding. The negative likelihood ratio remained close to 1 (LR − 0.68–0.80), confirming that a negative AI result did not meaningfully reduce the probability of crowding. For spacing detection at ± 1.0 mm, the F1 score was low (0.227) and the negative likelihood ratio was 0.84, again reflecting the platform’s inability to exclude true spacing (LR + 15.6, 95% CI 5.6–43.9, reflecting high specificity but very low sensitivity).

**Table 1:** Diagnostic accuracy of AI crowding detection at the tooth-pair level across five mLII thresholds (*N* = 875 tooth pairs, 88 patients)

Threshold (mm)	Prev. *n*	Prev. %	TP	FP	FN	TN	Sens. (95% CI)	Spec. (95% CI)	PPV (95% CI)	NPV (95% CI)	F1	LR+ (95% CI)	LR− (95% CI)	κ
± 0.5	379	43.3%	109	55	270	441	28.8% (24.4–33.5%)	88.9% (85.8–91.4%)	66.5% (58.9–73.2%)	62.0% (58.4–65.5%)	0.401	2.59 (1.93–3.48)	0.80 (0.75–0.86)	0.153
± 1.0	225	25.7%	84	80	141	570	37.3% (31.3–43.8%)	87.7% (84.9–90.0%)	51.2% (43.6–58.8%)	80.2% (77.1–82.9%)	0.432	3.03 (2.32–3.96)	0.71 (0.64–0.79)	0.247
± 1.5	119	13.6%	50	114	69	642	42.0% (33.5–51.0%)	84.9% (82.2–87.3%)	30.5% (24.0–37.9%)	90.3% (87.9–92.3%)	0.353	2.79 (2.13–3.65)	0.68 (0.58–0.80)	0.211
± 2.0	69	7.9%	27	137	42	669	39.1% (28.5–50.9%)	83.0% (80.3–85.4%)	16.5% (11.6–22.9%)	94.1% (92.1–95.6%)	0.232	2.30 (1.65–3.21)	0.73 (0.61–0.89)	0.129
± 2.5	35	4.0%	12	152	23	688	34.3% (20.8–50.8%)	81.9% (79.2–84.4%)	7.3% (4.2–12.4%)	96.8% (95.2–97.8%)	0.121	1.89 (1.17–3.06)	0.80 (0.63–1.02)	0.061

*Prev* prevalence, *TP* true positive, *FP* false positive, *FN* false negative, *TN* true negative, *Sens* sensitivity, *Spec.* specificity, *PPV* positive predictive value, *NPV* negative predictive value, *κ* Cohen’s kappa LR+, positive likelihood ratio, *LR−* negative likelihood ratio, *CI* confidence interval. F1 = 2TP / (2TP + FP + FN). The best operating point (± 1.0 mm) is highlighted

### Per-pair analysis

**Fig. 3:**
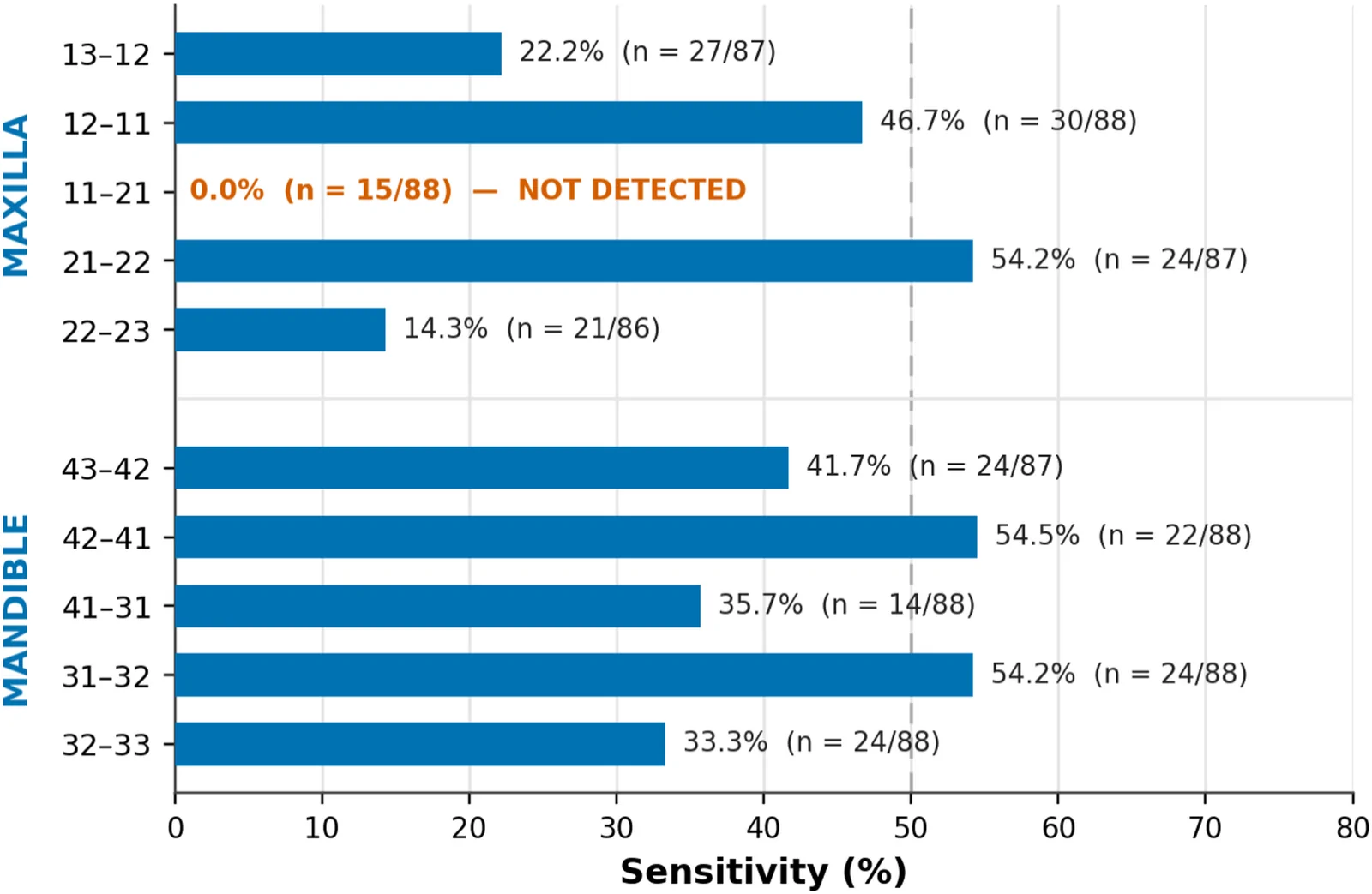
AI crowding sensitivity by Anterior Tooth Pair (mLII ≥ 1.0 mm)

At the ± 1.0 mm threshold, sensitivity varied markedly between tooth pairs (Table 2; Fig. 3). The highest sensitivities occurred at pairs 21–22, 42–41, and 31–32 (54.2–54.5%), and the lowest at pairs 22–23 (14.3%) and 13–12 (22.2%). A complete blind spot was observed at the 11–21 pair (the maxillary central incisors): despite 15 cases with mLII ≥ 1.0 mm, the AI flagged none (sensitivity 0.0%), and this pattern was consistent across all thresholds. Mandibular pairs showed higher overall sensitivity (44.4%) than maxillary pairs (30.8%).

**Table 2:** Per-pair crowding detection at the ± 1.0 mm threshold

Tooth pair	*N*	Mean mLII	Crowded (ref)	AI crowding	True Positive	Sensitivity	Specificity
13–12	87	0.651	27	8	6	22.2%	96.7%
12–11	88	0.746	30	18	14	46.7%	87.9%
11–21	88	0.297	15	0	0	0.0%	100%
21–22	87	0.625	24	16	13	54.2%	87.3%
22–23	86	0.494	21	7	3	14.3%	89.2%
43–42	87	0.557	24	12	10	41.7%	90.5%
42–41	88	0.495	22	17	12	54.5%	78.8%
41–31	88	0.121	14	6	5	35.7%	86.5%
31–32	88	0.496	24	17	13	54.2%	75.0%
32–33	88	0.508	24	15	8	33.3%	84.4%

*mLII* modified Little’s Irregularity Index,*ref* reference

### Spacing detection

Spacing detection was consistently poor. At the ± 1.0 mm threshold, only 5 of 30 spacing cases were detected (sensitivity 16.7%; 95% CI 7.3–33.6%). Specificity was high (98.9%) and PPV was 35.7%, so that when the AI flagged spacing it was correct in roughly one-third of cases. The great majority of true spacing was missed (false-negative rate 83.3%). The small number of spacing events (8–69, depending on the threshold) limits the precision of these estimates.

### Arch-level analysis

At the arch-segment level (176 arches from 88 patients), the mean total LII was 3.55 ± 3.25 mm (range 0.00–17.00 mm). The grade distribution was as follows: perfect alignment, 17 arches (9.7%); minimal, 80 (45.5%); moderate, 42 (23.9%); severe, 29 (16.5%); and very severe, 8 (4.5%).

**Fig. 4:**
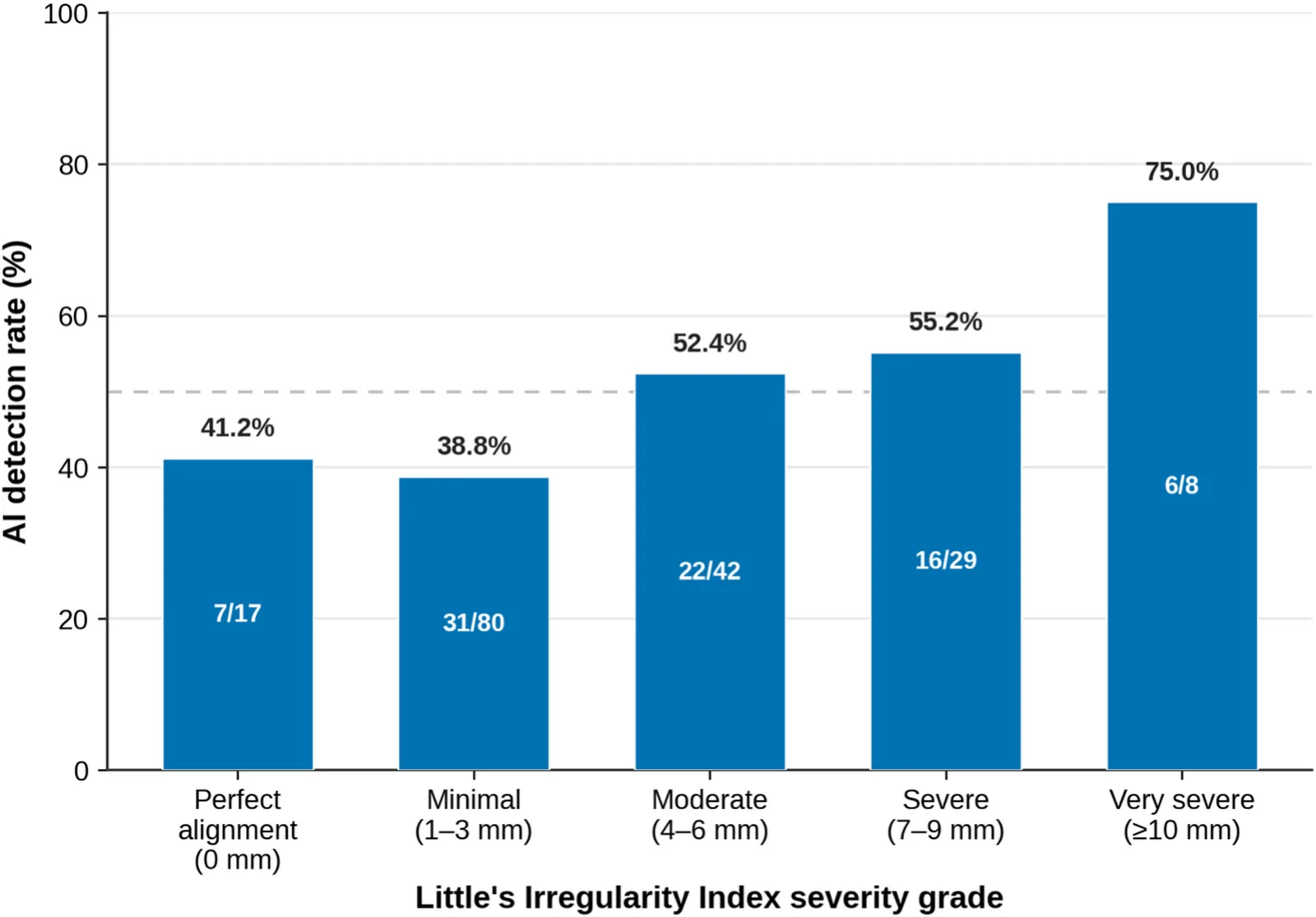
AI anterior crowding detection rate by Little’s Irregularity Index Severity Grade

**Fig. 5:**
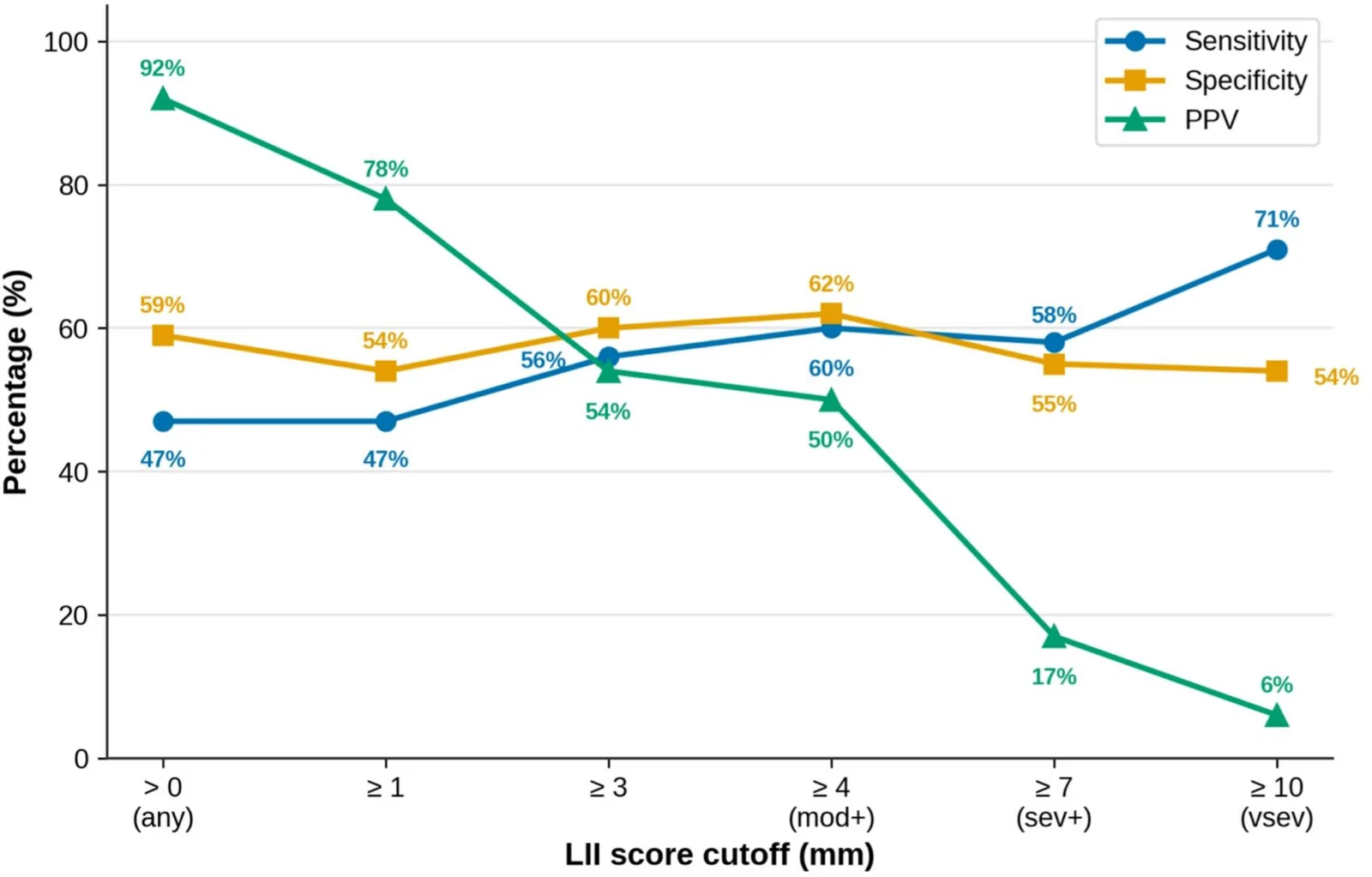
Arch-level crowding detection: AI performance across Little’s Irregularity Index (LII) severity cut-offs. At each cut-off, arches were dichotomized as crowded (total LII ≥ cut-off) or aligned, and the AI diagnosis (any anterior tooth flagged as crowded) was compared against this reference. Sensitivity, specificity, and Cohen’s κ are plotted; higher cut-offs increased sensitivity at the expense of specificity. The best balance was obtained at the ≥ 4 mm cut-off (sensitivity 60.3%, specificity 62.0%, κ = 0.215)

Arch-level sensitivity increased from 47.2% (LII > 0 mm) to 71.4% (LII ≥ 10 mm), confirming that the AI was more likely to detect crowding in more severely affected arches (Fig. 4). This severity-dependent trend was clear, with detection rising from 38.8% for minimal irregularity to 75.0% for very severe irregularity. The best balance between sensitivity and specificity was obtained at the ≥ 4 mm cut-off (sensitivity 60.3%, specificity 62.0%, κ = 0.215). However, the AI also flagged crowding in 41.2% of perfectly aligned arches (LII = 0 mm), indicating a substantial false-positive rate (Fig. 5).

### Summary of key findings

The Diagnocat platform showed limited sensitivity for detecting anterior crowding at both the tooth-pair level (28.8–42.0%) and the arch-segment level (47.2–71.4%), with a clear severity-dependent trend. When the AI flagged crowding, the diagnosis was usually correct at lenient thresholds (PPV 66.5% at ± 0.5 mm) but unreliable at stringent thresholds (PPV 7.3% at ± 2.5 mm). A systematic blind spot was identified at the 11–21 pair (sensitivity 0%). Spacing detection was poor across all thresholds (sensitivity 10–25%). Overall agreement with the mLII reference was fair (κ = 0.247 at the tooth-pair level; κ = 0.215 at the arch level).

## Discussion

This study evaluated the accuracy of an AI system for detecting anterior dental crowding and spacing on CBCT, using a dual reference framework: the modified Little’s Irregularity Index (mLII) at the tooth-pair level and the conventional LII at the arch level. The AI showed limited sensitivity and only fair agreement with the reference, missing a substantial proportion of clinically relevant irregularities at the tooth-pair level. Because this deficit was consistent across all five thresholds, it reflects a fundamental limitation of the platform rather than an artefact of any single threshold. The most striking finding was the systematic failure at the maxillary central-incisor pair (11–21), where no crowded case was identified despite confirmed displacement — a clinically important blind spot in one of the most visible and patient-relevant regions of the dentition. At the arch level, detection improved with severity but still missed a quarter of the most severe cases and produced false positives in perfectly aligned arches.

Modern AI systems segment teeth on CBCT with high accuracy [9, 13], and in theory, the spatial relationships between segmented crowns should reveal crowding and spacing directly. A recent publication showed that at the tooth level, the Diagnocat platform is highly accurate in tooth segmentation in both CBCT and panoramic images [30]. Our findings indicate that accurate segmentation does not automatically translate into accurate detection of contact-point discrepancies: the platform’s per-pair classification appears to rely on internal decision rules that are not fully aligned with the contact-point geometry on which clinical indices such as the LII are based. Equally important is the choice of imaging modality. Crowding and spacing are defined by contact-point relationships that are most directly and accurately captured by intraoral scans or dental casts, and AI tools that segment teeth on digital scans have achieved high accuracy for crowding and spacing [31, 32]. CBCT, by contrast, is acquired primarily for skeletal, alveolar, and impacted-tooth assessment, delivers a higher radiation dose, and is susceptible to artefacts that can obscure the very contact points required for this measurement. The limited performance observed here should therefore be understood partly as a mismatch between the diagnostic task and the imaging modality, and not solely as a deficiency of the algorithm. In clinical practice, crowding and spacing should continue to be assessed from clinical examination and intraoral scans, with CBCT reserved for indications that genuinely require three-dimensional imaging [33].

To our knowledge, this is the first study to evaluate AI-based crowding and spacing detection on CBCT at the level of the individual tooth pair. A recent investigation of the same platform examined skeletal pattern, vertical classification, and Angle classification on CBCT and found that the software failed to generate a diagnosis in more than 90% of skeletal and vertical assessments and achieved only fair agreement (κ = 0.31) for dental Angle classification [34]. The authors concluded that the platform’s diagnostic reliability was insufficient for CBCT-based categorical orthodontic reporting, largely because of the very low completeness of its skeletal and vertical outputs. Together with the present results, this provides complementary evidence that the current version of the Diagnocat orthodontic module does not meet the threshold for standalone clinical use across its diagnostic domains.

Direct comparison with the wider literature is limited, because few studies have assessed AI-based crowding detection on CBCT at the per-tooth level; the available evidence spans purpose-built deep-learning models applied to intraoral photographs, general-purpose large language models (LLMs) applied to occlusal images, and AI-integrated software applied to 3D digital models [16, 31, 32, 35, 36]. Among purpose-built systems, Hatoum et al. [31] validated a YOLOv8-Pose model for tooth size–arch length discrepancy on intraoral photographs against OrthoCAD measurements, achieving 90% accuracy at a 2 mm clinical threshold and ICCs of 0.948 for crowding and 0.920 for spacing. Wang et al. [35] developed DCC-Net, an explainable deep-learning system trained on more than 2,800 intraoral photographs, which reached sensitivities of 67.9% and 67.2% for maxillary and mandibular crowding and improved the diagnostic accuracy of junior orthodontists. Hertig et al. [36] trained a CNN to quantify mandibular anterior crowding from single occlusal photographs and reported excellent reliability for both contact-point displacement (ICC = 0.900; mean absolute difference [MAD] = 0.36 mm) and aggregate LII (ICC = 0.930; MAD = 0.74 mm). These results contrast sharply with our findings for Diagnocat (sensitivity 28.8–42.0%; maximum κ = 0.247). The contrast most likely reflects fundamental differences in design: these systems were purpose-built, trained on annotated intraoral records, and produced continuous quantitative output, whereas Diagnocat is a general-purpose commercial platform that operates on CBCT and reports only qualitative per-pair labels.

Evidence from other AI paradigms points in the same direction. Wafaie et al. [16] evaluated three commercial generative LLMs (ChatGPT-4o mini, Microsoft Copilot, and Claude 3.5 Sonnet) for classifying crowding severity from occlusal photographs; the best-performing model classified only half of the images correctly, and all three combined high specificity with low sensitivity and limited repeatability — a pattern that closely mirrors our own. In the 3D-software domain, Bor et al. [32] compared Titan Dental Design and SoftSmile with clinician measurements on intraoral-scan models and found almost perfect agreement for Angle molar classification but statistically significant discrepancies for overjet, overbite, and space analysis (*p* < 0.001), which they attributed largely to difficulty in identifying mesial and distal contact points. The recurrence of contact-point identification as a failure mode across modalities reinforces our interpretation that this geometric step, rather than tooth segmentation itself, is the principal bottleneck for automated crowding and spacing assessment.

**Fig. 6:**
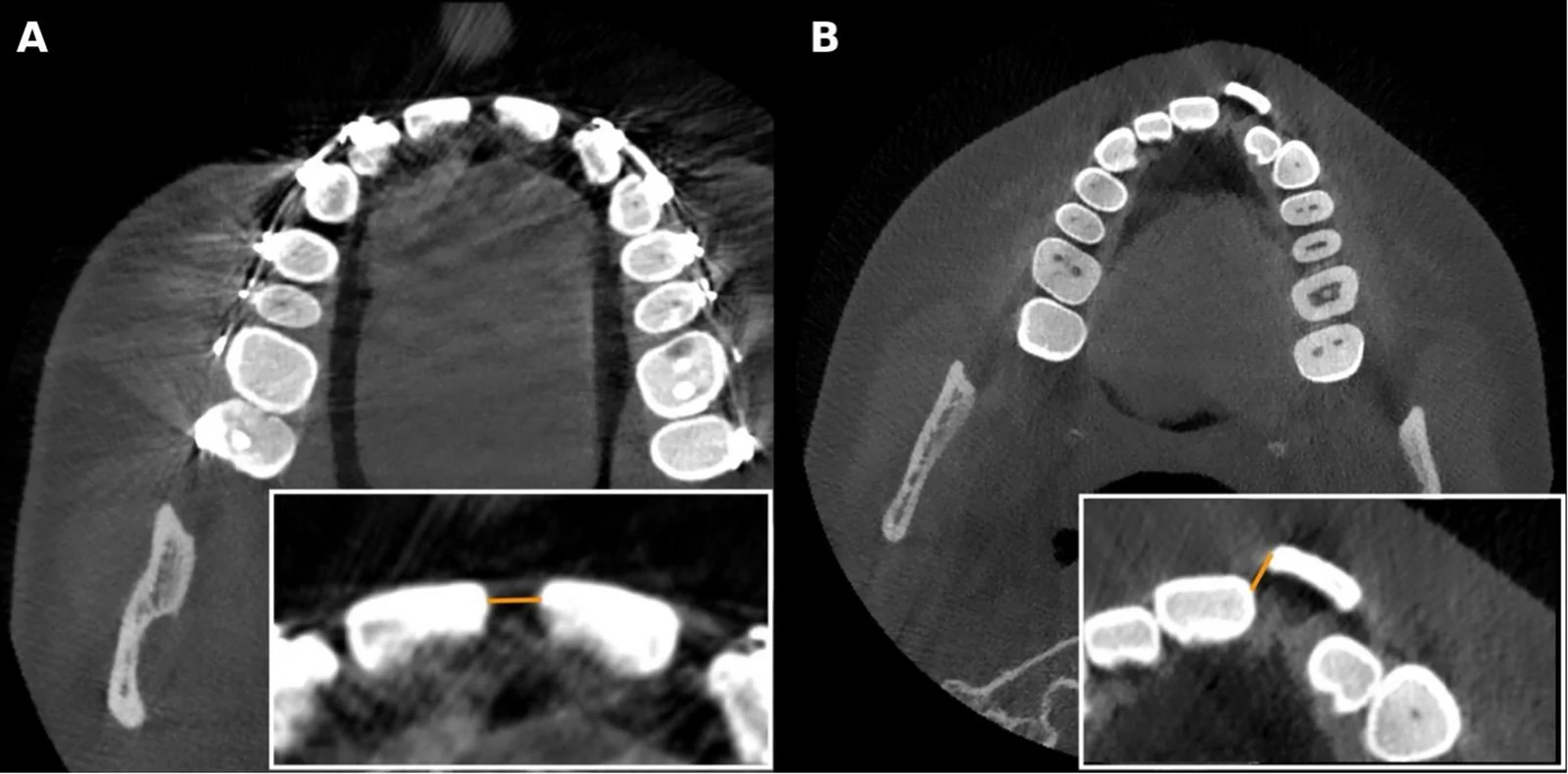
Two of the 15 false-negative cases involving tooth crowding/spacing in the 11–21 tooth pair. (**A**) An 18-year-old female with an mLII consensus value of − 2.8 mm (orange line). (**B**) A 17-year-old male with severe crowding and an mLII consensus value of 3.55 mm (orange line)

The complete blind spot at the 11–21 pair warrants particular attention. The AI detected none of the 15 cases with mLII ≥ 1.0 mm at this location and, indeed, did not diagnose a single instance of crowding or spacing here in the entire cohort, despite some severe cases (Fig. 6). Several mechanisms may contribute. The maxillary midline is a region of near-bilateral symmetry, where the two central incisors are anatomically very similar; this may introduce localisation or labelling ambiguity, and segmentation may be less stable where the two crowns meet at the midline. It is also possible that the platform applies a built-in assumption of midline symmetry that suppresses discrepancy detection at this junction. Whatever the underlying mechanism, the clinical consequence is significant, because diastema and crowding between the maxillary central incisors are among the most common orthodontic concerns and frequently prompt patients to seek treatment. This observation echoes those of Hatoum et al. [31], who found the largest discrepancies in specific discrepancy categories, and of Bor et al. [32], whose contact-point errors propagated into downstream measurements.

At the arch level, AI detection increased with crowding severity, from 38.8% for minimal irregularity to 75.0% for very severe irregularity. Even in the most severe category, however, a quarter of cases were missed, so a negative AI report cannot be taken as evidence that clinically relevant crowding is absent. Conversely, the AI flagged crowding in 41.2% of perfectly aligned arches, generating a substantial false-positive burden, and the best overall agreement (κ = 0.215, at the ≥ 4 mm cut-off) remained only fair. Spacing detection was poorer still (sensitivity 10.1–25.0%): specificity exceeded 98.6%, but the very low sensitivity shows that the platform essentially fails to identify anterior spacing. Although the small number of spacing cases limits statistical precision, the consistency of this pattern across thresholds argues for a genuine limitation rather than a chance finding. Hatoum et al. [31] likewise reported somewhat lower AI accuracy for spacing than for crowding, although their purpose-built model performed substantially better overall than the general-purpose platform evaluated here.

These findings have several practical implications for clinicians, particularly within the European regulatory context. First, a “no crowding” report from a CBCT AI platform does not reliably exclude clinically significant anterior irregularity and should not be used to triage patients away from clinician review. Second, the maxillary midline (the 11–21 region) warrants routine manual verification in every CBCT AI report, because the blind spot identified here may persist across broader case mixes. Third, under the EU Artificial Intelligence Act (Regulation (EU) 2024/1689), which has been in force since 2024 and classifies clinical AI as high-risk, responsibility for diagnostic decisions remains with the clinician; independent, task-specific validation of the kind reported here is increasingly relevant to manufacturers’ post-market surveillance obligations. Finally, the high PPV observed at lenient thresholds indicates that a positive AI flag can still serve as a useful screening alert: it should prompt focused clinician review of the flagged region rather than be treated as a confirmatory diagnosis. Crucially, because crowding and spacing are most accurately assessed from clinical examination and intraoral records, and because CBCT carries a radiation burden, CBCT-based AI should not be regarded as the appropriate route to this diagnosis in the first place.

This study has several strengths. The reference standard was validated and showed excellent inter-reader reliability (ICC = 0.946; MAD 0.345 mm). Diagnostic accuracy was characterised across five thresholds, enabling a comprehensive sensitivity analysis, and the dual-level design allowed interpretation at both the tooth-pair and arch granularities. The analysis was based on a large number of observations (875 tooth pairs and 176 arches), and reporting followed the STARD guidelines, enhancing transparency. To our knowledge, this is also the first evaluation of AI crowding and spacing detection on CBCT at the level of the individual tooth pair, addressing a gap highlighted by recent reviews [14, 15].

Several limitations should be acknowledged. First, the mLII reference standard was confined to the anterior segment (canine to canine), so the accuracy of the AI for posterior crowding and spacing could not be assessed. Second, the signed mLII is a methodological adaptation developed specifically for the purposes of the present validation study rather than a new, independently validated index. It was introduced solely to preserve directional information (crowding versus spacing) and to align the reference standard with the per-pair output format of the AI platform; the underlying geometric measurement — linear contact-point displacement on standardized axial slices — is identical to the validated LII. The mLII is therefore not proposed as a stand-alone diagnostic instrument, and independent validation of the signed, per-pair convention in separate cohorts would be required before it is applied beyond the context of this study. Third, the study population was drawn from three private clinics in a single country, which may limit generalisability. Fourth, only one commercial platform was evaluated, so the results cannot be extrapolated to other AI tools or to purpose-built deep-learning models applied to CBCT; a head-to-head comparison was beyond the scope of this work, which was designed to characterise the per-pair performance of one of the most widely deployed commercial CBCT modules. Fifth, a single software version was assessed, and performance may change as the algorithm is updated. Sixth, we had no access to the platform’s internal thresholds or architecture, which prevents a fully mechanistic explanation of the observed patterns. Finally, the retrospective design carries a risk of selection bias, because patients who undergo CBCT may not be representative of the general orthodontic population.

Future work should extend the mLII to larger, multicentre samples and evaluate its validity for posterior segments. Comparative studies that assess several AI platforms against a common reference standard would help clinicians choose between tools, and longitudinal studies tracking performance across software versions would document how diagnostic accuracy evolves. Given that intraoral scans and dental casts are the natural records for this task, future research should also test whether purpose-built deep-learning architectures, such as YOLO-based approaches [31], can be adapted to CBCT or, preferably, integrated into intraoral-scan workflows for crowding and spacing assessment.

## Conclusions

The Diagnocat AI platform showed limited sensitivity and only fair agreement for detecting anterior crowding and spacing on CBCT and should not be used as a standalone diagnostic tool for this purpose. A “no crowding” report does not reliably exclude clinically significant anterior irregularity, and the complete blind spot at the maxillary central-incisor pair is a particular concern that should be addressed in future software versions. More fundamentally, crowding and spacing are most reliably assessed from clinical examination and intraoral records; given the radiation involved, CBCT — and CBCT-based AI in particular — should not be relied upon for this diagnosis. Finally, AI outputs should support, not replace, or bias clinician judgement.

## Data Availability

The de-identified datasets supporting the findings of this study, including the modified Little’s Irregularity Index (mLII) measurements, AI platform output classifications, and summary statistics, are available from the corresponding author (w.kazimierczak@cm.umk.pl) upon reasonable request. Raw DICOM files cannot be shared publicly owing to patient privacy obligations and data protection regulations, even though they are fully de-identified, as they contain detailed three-dimensional imaging data that could theoretically pose re-identification risks.

## Abbreviations

2D: Two-dimensional
3D: Three-dimensional
AI: Artificial Intelligence
CBCT: Cone-beam Computed Tomography
CI: Confidence Interval
CNN: Convolutional Neural Network
DCC-Net: Dental Crowding Categorization Network
DICOM: Digital Imaging and Communications in Medicine
FN: False Negative
FOV: Field of View
FP: False Positive
ICC: Intraclass Correlation Coefficient
LII: Little’s Irregularity Index
LLM: Large Language Model
MAD: Mean Absolute Difference
mLII: Modified Little’s Irregularity Index
MPR: Multiplanar Reconstruction
NPV: Negative Predictive Value
PACS: Picture Archiving and Communication System
PPV: Positive Predictive Value
SD: Standard Deviation
STARD: Standards for Reporting of Diagnostic Accuracy Studies
TN: True Negative
TP: True Positive
